# Topographic location of unisolated pontine infarction

**DOI:** 10.1186/s12883-019-1411-6

**Published:** 2019-08-05

**Authors:** Jiehong Huang, Zhihua Qiu, Piansi Zhou, Jianming Li, Yingxin Chen, Ruiyun Huang, Chujing Li, Xilin Ouyang, Huazhuo Feng, Hanqing Xu, Dezhi Liu, Zheng Dai, Juehua Zhu, Xinfeng Liu, Hongbing Chen, Yongjun Jiang

**Affiliations:** 1 Department of Neurology, The Second Affiliated Hospital of Guangzhou Medical University, 250 Changgang East Road, Guangzhou, 510260 China; 2 Department of Neurology, Shanghai General Hospital, Shanghai Jiao Tong University School of Medicine, Shanghai, 200080 China; 3 Department of Neurology, Wuxi People’s Hospital, 299 Qingyang Road, Wuxi, 214023 China; 4 Department of Neurology, The First Affiliated Hospital of Soochow University, 899 Pinghai Road, Suzhou, 215300 China; 5 Department of Neurology, Jinling Hospital, Medical School of Nanjing University, 305 Zhongshan East Road, Nanjing, 210002 China; 6 Department of Neurology and Stroke Center, The First Affiliated Hospital, Sun Yat-Sen University, 58 Zhongshan Road II, Gungzhou, 510080 China

**Keywords:** Stroke, Pontine infarction, Isolated pontine infarction, Unisolated pontine infarction, And topographic location

## Abstract

**Background:**

The topographic location of acute pontine infarction is associated with clinical syndromes and prognosis. Previous studies focused on isolated pontine infarction, but the topographic location of unisolated pontine infarction has remained unclear.

**Methods:**

This was a prospective, multicenter, longitudinal registry study. Patients with acute pontine infarction confirmed by magnetic resonance imaging (MRI) were enrolled. Based on the territory of the pontine artery, the topographic location was divided into anteromedial, anterolateral, tegmental, bilateral and unilateral multiple infarctions.

**Results:**

From May 1, 2003, to Oct 31, 2017, 1003 patients were enrolled, and 330 had unisolated pontine infarction. For isolated pontine infarction, 44.9, 19.8, 16.0, 13.1 and 6.2% of patients had anteromedial, anterolateral, tegmental, bilateral and unilateral multiple pontine infarctions, respectively. For unisolated pontine infarction, 30.3, 19.7, 24.5, 15.2 and 10.3% of patients had anteromedial, anterolateral, tegmental, bilateral and unilateral multiple pontine infarctions, respectively.

**Conclusion:**

In this large series study, our data revealed fewer anteromedial infarctions and more tegmental and unilateral multiple infarctions in patients with unisolated pontine infarction than in patients with isolated pontine infarction.

## Background

Pontine infarction is the most common type of stroke in the posterior circulation territory and accounts for 7% of all ischemic strokes [1, 2]. There are two kinds of pontine infarction based on the presence of infarction in other brain areas: isolated and unisolated pontine infarctions.

In brief, previous studies usually recruited patients with isolated pontine infarction [1, 3–6]. In the 1960s, Miller Fisher described the clinical syndromes of isolated pontine infarction, including pure motor hemiparesis, dysarthria-clumsy hand, ataxic hemiparesis, and homolateral ataxia with crural paresis [7]. In the following decades, the topographic location of isolated pontine infarction and its role were thoroughly investigated [3, 5, 6, 8–11]. Semi et al. found that the topographic location of acute pontine infarction was associated with progressive motor deficits [5]. Long-term follow-up revealed that ventral infarcts had a less favorable prognosis than anteromedial, tegmental and lateral pontine infarcts did [9]. Kazunori et al. investigated the topographic location of pontine infarction with extrapontine infarct in the posterior circulation in a small series [12]. As magnetic resonance imaging (MRI) is popular in clinical practice, the correlation of neurological deficits with topographic location has been thoroughly investigated [13].

Although unisolated pontine infarction is often identified in clinical practice [14], there is an absence of data about its topographic location. Thus, we aimed to investigate the topographic location of unisolated pontine infarction in a large series study.

## Methods

### Patients

The research protocol [15] was reviewed and approved by the ethics committees of each institute (Guangzhou Medical University, Jinling Hospital, Sun Yat-Sen University, The First Affiliated Hospital of Soochow University, Wuxi People’s Hospital and Shanghai General Hospital). Written consent was obtained from patients or their authorized relatives before enrollment.

Data were prospectively collected from patients with acute pontine infarction. The patients were enrolled if they met the inclusion criteria as follows: (1) older than 18 years; (2) admitted to the hospital within 7 days of stroke onset; (3) MRI (including diffusion-weighted imaging, DWI) and magnetic resonance angiogram (MRA); and (4) intracranial and extracranial cerebral arteries visualized by ultrasound, MRA, computed tomography angiography (CTA) or digital subtraction angiography (DSA). The patients were excluded if they met any of the following criteria: (1) missing clinical or imaging information, (2) brain tumor, (3) brain parasites, or (4) no lesion in DWI images.

### Clinical information

On admission, demographic information (including age and gender), stroke risks (current smoker, current drinker, hypertension, hyperlipidemia, diabetes, coronary artery disease, and previous stroke or transient ischemic attack (TIA) history) and the National Institutes of Health Stroke Scale (NIHSS) on admission were collected. Current drinkers were categorized by heavy intake (more than 14 drinks per week in women or more than 21 drinks per week in men) or episodic heavy intake (more than 5 drinks in 1 episode at least once per month) [16]. Blood tests (complete blood count, biochemistry, and coagulation profile) and cardiac examinations (electrocardiogram and echocardiogram) were also performed. Atrial fibrillation (AF) was diagnosed based on the medical history, symptoms, signs and electrocardiogram (ECG).

### MRI and MRA

All patients underwent MRI on admission using either a 1.5 T or a 3.0 T MRI unit. Scanning sequences included T1-weighted imaging, T2-weighted imaging, fluid-attenuated inversion recovery sequence, apparent diffusion coefficient maps, DWI and time-of-flight MRA covering the circle of Willis. The stroke subtype was determined based on the DWI images with reference to other sequences.

**Fig. 1:**
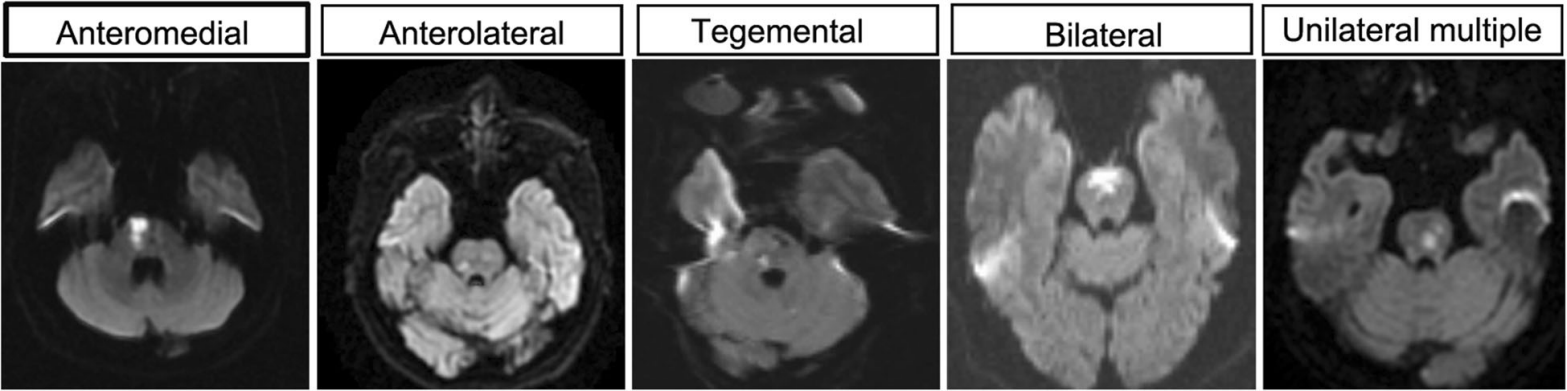
Topographic location of pontine infarction. There were 5 subtypes of pontine infarction: anteromedial, anterolateral, tegmental, bilateral and unilateral multiple pontine infarctions

**Fig. 2:**
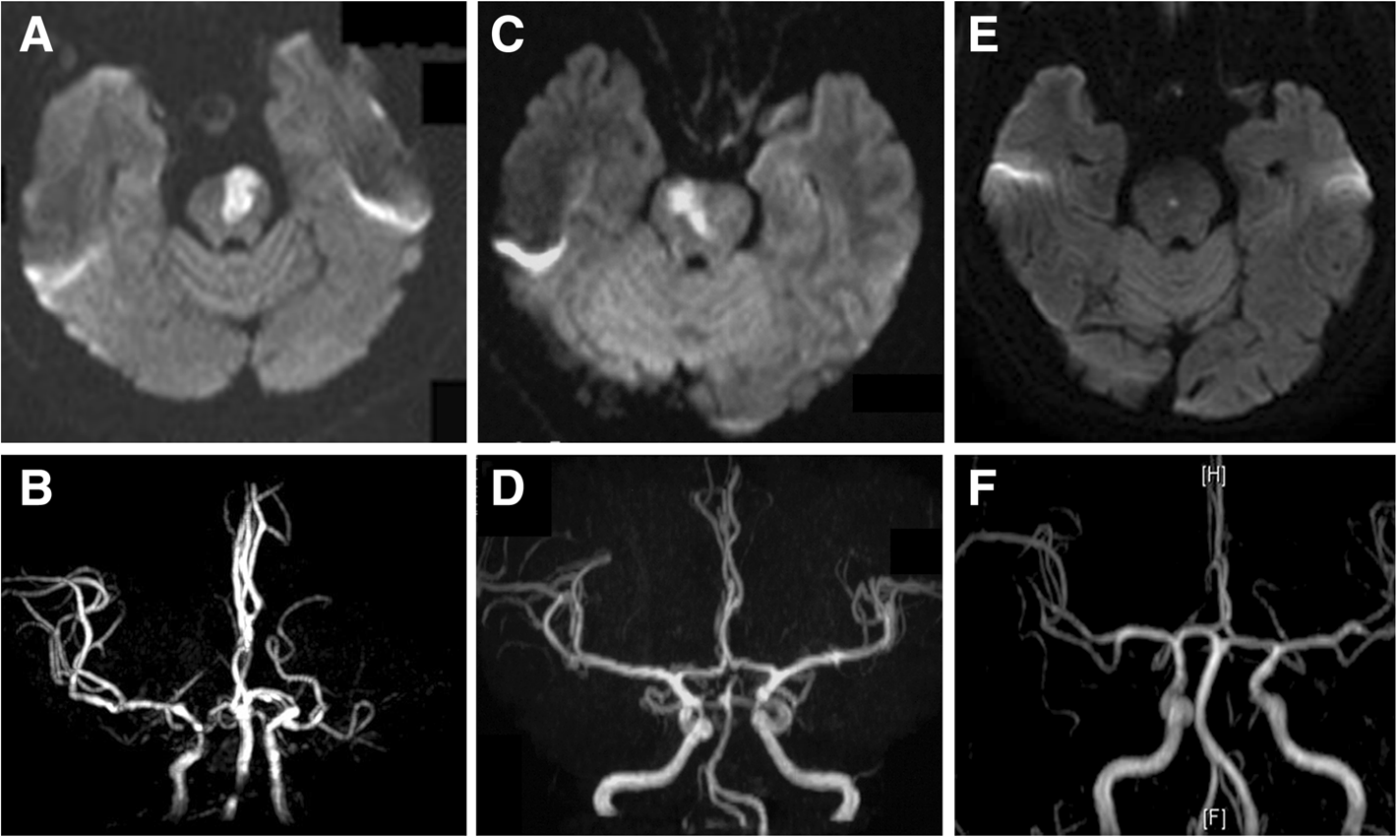
Mechanism of pontine infarction. **a** LAD and (**b**) MRA of LAD with significant BA stenosis; (**c**) BABD and (**d**) MRA; (**e**) SAD and (**f**) MRA

Based on the territory of the pontine artery, pontine infarction has 5 subtypes (Fig. 1): anteromedial, anterolateral, tegmental, bilateral and unilateral multiple pontine infarctions [9]. The anteromedial pontine is supplied by the anteromedial pontine artery (foramen coecum arteries, paramedian pontine arteries, and interpeduncular fossa arteries). The anterolateral pontine is supplied by the anterolateral pontine artery. The tegmentum pontine is supplied by the lateral pontine arteries (anterior inferior cerebellar artery and superior cerebellar artery) and posterior pontine arteries (medial and lateral branches of the superior cerebellar artery) [8]. The section with the largest area of infarction in the rostrocaudal direction was used to evaluate whether the lesion was anteromedial, anterolateral or tegmental. All sections in the rostrocaudal direction and coronal direction were evaluated to determine whether the lesion was bilateral or unilateral multiple. The mechanism of pontine infarction was divided into 3 kinds according to previous studies [11]: large artery disease (LAD), basilar artery branch disease (BABD) and small artery disease (SAD). (1) LAD: pontine infarction with basilar artery (BA) stenosis or occlusion corresponding to the infarcts; (2) BABD: infarcts that reach or approach the basal surface of the ventral pontine without BA stenosis corresponding to the infarcts; (3) SAD: deeper infarcts without the involvement of the ventral surface in the absence of BA stenosis in areas corresponding to the infarcts (Fig. 2). If patients had recurrent pontine infarction, MRI images of the first stroke onset were used. BA stenosis was defined as the presence of stenosis in > 50% of the BA on MRA.

### Statistical analysis

Continuous variables are expressed as the mean ± standard deviation, and categorical variables are expressed as percentages. The differences in continuous variables were compared with the homogeneity test of variances. Student’s *t*-test or analysis of variance (ANOVA) was used when normality assumptions were met; otherwise, the equivalent nonparametric test was used. Pearson’s Chi-square test was used to analyze categorical variables. To compare the difference in topographic location between isolated and unisolated pontine infarction, we used Pearson’s Chi-square test with post hoc analysis. *P* < 0.05 was considered statistically significant. SPSS 20.0 was used for statistical analysis.

## Results

### Patient profile

**Fig. 3:**
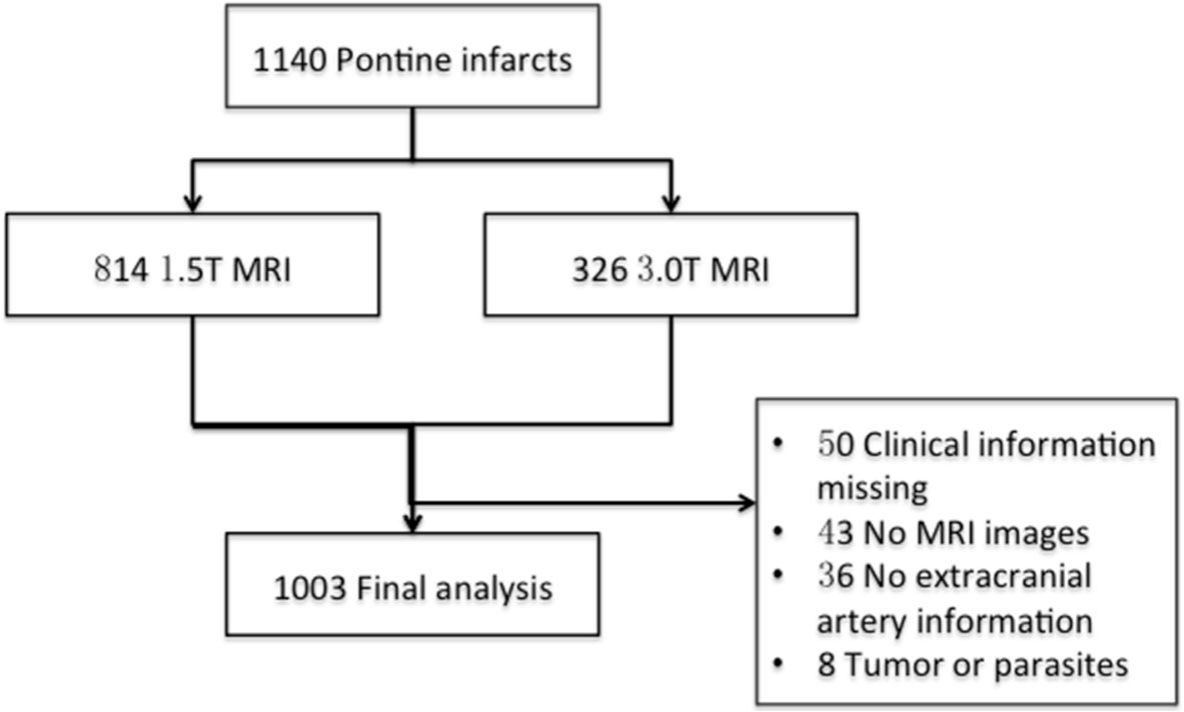
Flow diagram

From May 1, 2003, to Oct 31, 2017, a total of 1140 patients with acute pontine infarction were screened (450 from The Second Affiliated Hospital of Guangzhou Medical University, 144 from Jinling Hospital, 326 from Sun Yat-Sen University, 101 from The First Affiliated Hospital of Soochow University, 72 from Wuxi People’s Hospital and 47 from Shanghai General Hospital), and 1003 patients were enrolled in the analysis (Fig. 3). A total of 673 patients had isolated pontine infarction, and 330 had unisolated pontine infarction. The clinical characteristics are presented in Table 1. AF was more prevalent in patients with unisolated pontine infarction than in those with isolated pontine infarction (7.6% vs 3.0%, *P* < 0.001).

**Table 1:** Baseline characteristics

Characteristics	Isolated pontine infarction	Unisolated pontine infarction	*P*
Number	673	330	
Age (yrs)	62.7 ± 13.1	62.6 ± 13.4	0.947
Male	60.1%	63.6%	0.291
Hypertension	79.9%	78.5%	0.592
Diabetes	37.3%	36.7%	0.846
CHD	8.8%	11.2%	0.216
AF	3.0%	7.6%	0.001
High TG	38.8%	32.1%	0.040
High TC	40.5%	30.6%	0.002
High LDL	30.2%	24.8%	0.079
Uric acid	19.3%	20.6%	0.630
Smoker	28.7%	31.2%	0.408
Drinker	18.7%	17.6%	0.659
Medication			
Antihypertensive	63.3%	62.1%	0.717
Hypoglycemic	35.2%	33.3%	0.556
Statins	71.6%	71.2%	0.893
Antiplatelet			0.219
ASA	47.1%	53.0%	0.085
Clo	36.4%	31.8%	0.152
DAPT	16.3%	15.2%	0.691
Anticoagulation	6.7%	8.2%	0.389
Previous stroke or TIA	25.0%	23.9%	0.724
Previous pontine infarction	16.9%	18.5%	0.544
NIHSS	5.34 ± 3.16	5.34 ± 3.59	0.992
BA stenosis	44.8%	44.5%	0.922
Topographic location			< 0.001
Anterolateral	19.8%	19.7%	0.981
Anteromedial	44.9%	30.3%	< 0.001
Tegmental	16.0%	24.5%	0.001
Bilateral	13.1%	15.2%	0.370
Unilateral multiple	6.2%	10.3%	0.022
Mechanism			< 0.001
LAD	16.5%	24.5%	0.002
BABD	49.2%	32.4%	< 0.001
SAD	34.3%	43.0%	0.007

*CHD* coronary heart disease, *AF* atrial fibrillation, *TG* triglyceride, *TC* total cholesterol, *LDL* low density lipoprotein, *ASA* aspirin, *Clo* clopidogrel, *DAPT* dual antiplatelet therapy, *TIA* transient ischemic attack, *NIHSS* The National Institutes of Health Stroke Scale, *BA* basilar artery, *LAD* large artery disease, *BABD* basilar artery branch disease; SAD, small artery disease

### Topographic location of pontine infarction

As shown in Table 1, there was a significant difference in the topographic location between isolated and unisolated pontine infarction. Unisolated pontine infarction had more unilateral multiple infarction (10.3% vs 6.2%, *P* = 0.022) and tegmental infarction (24.5% vs 16.0%, *P* < 0.001). Isolated pontine infarction had more anteromedial pontine infarction (44.9% vs 30.3%, *P* = 0.000), although it was the most common subtype for both isolated and unisolated pontine infarction. Anterolateral and bilateral pontine infarctions were similar between isolated and unisolated pontine infarctions.

### Mechanism of pontine infarction

The mechanism of pontine infarction was classified into LAD, BABD and LAD. For isolated pontine infarction, BABD was the leading cause (49.2%, *P* = 0.002, Table 1). For unisolated pontine infarction, SAD was the leading cause (43.0%, *P* = 0.000, Table 1). The topographic location was significantly related to the mechanism (Table 2).

**Table 2:** Topographic location of pontine infarction

Topographic location	LAD	BABD	SAD
Anterolateral	32	63	103
Anteromedial	78	250	74
Tegmental	26	0	163
Bilateral	32	80	26
Unilateral multiple	24	44	8

## Discussion

The main findings of our present study are as follows: (1) pontine infarction (including isolated and unisolated pontine infarctions) was mostly located in the ventral pontine, which was divided into anteromedial and anterolateral; (2) compared to isolated pontine infarction, unisolated pontine infarction had more unilateral multiple and tegmental infarctions but fewer ventral medial pontine infarctions; (3) most isolated pontine infarctions were caused by BABD, while most unisolated pontine infarctions were caused by SAD. At present, our study has the largest number of patients with acute pontine infarction.

Based on our data, pontine infarction (59.8%, 600/1003) was mostly located in the ventral pontine. Silverstein et al. found that 51% of infarctions were located in the ventral pontine in 81 autopsied cases. These autopsied cases may represent severe pontine infarction, which may result in selection bias. For isolated pontine infarction, Claudio et al. found that 58% of isolated pontine infarctions were located in the ventral pontine in an MRI-based study [8]. In another MRI-based study, 75% of isolated pontine infarctions had ventral infarctions [9]. These inconsistencies in prevalence are due to the small number of subjects in each individual study. In this large series study, 64.7% of isolated pontine infarctions were located in the ventral pontine. For unisolated pontine infarction, no previous studies provided any information, and we found that 50.0% of unisolated pontine infarctions were located in the ventral pontine. Ventral infarction was divided into anteromedial and anterolateral, and most ventral infarctions were located in the anteromedial part. Compared to isolated pontine infarction, unisolated pontine infarction had fewer anteromedial infarctions.

For the tegmental pontine, Claudio et al. showed that 31% of isolated pontine infarctions were located in the tegmental pontine [8], while Emre et al. reported only 12% [9]. According to our data, 16.0% of isolated pontine infarctions had tegmental infarction. For unisolated pontine infarction, there was 24.5% tegmental infarction. Tegmental infarction usually represents SAD, and most unisolated pontine infarctions were caused by SAD in our study. Bilateral pontine infarction indicates severe neurological dysfunction and is related to poor outcomes [17, 18]. Claudio et al. showed that 11% of isolated pontine infarctions were bilateral ventral infarctions [8]. In a study by Emre et al., 9.3% of isolated pontine infarctions were bilateral infarctions. Our data showed that 13.1% of isolated pontine infarctions were bilateral infarctions. Silverstein et al. thought that bilateral infarction might be related to other brainstem infarctions, which was not proven in our study. Here, the proportion of bilateral pontine infarction remained consistent among the different groups. A previous study showed that 4% of isolated pontine infarctions were unilateral multiple infarctions [9], consistent with our findings. For unisolated pontine infarction, the proportion of unilateral multiple infarction was 2 times higher.

The topographic location indicated the possible mechanism of pontine infarction. This was proved by the previous studies [1, 3] and ours. The different mechanism resulted in the different prognosis. The BABD was an independent risk for neurological deterioration in the acute phase of pontine infarction [1]. The LAD had the worst outcome followed by the BABD and SVD [11]. Our data showed that most of isolated pontine infarction was caused by BABD while most of unisolated pontine infarction was caused by SAD. This suggested that isolated and unisolated pontine infarction might have the different prognosis. This need the confirmation in the future studies.

Limitations First, this study was MRI based, and some patients who could not receive MRI scanning, such as those with pacemakers, were not enrolled. Second, the mechanism of pontine infarction was mostly diagnosed based on the topographic location, which might have caused some bias. Finally, we found that the mechanism of pontine infarction was related to the topographic location. However, the role of topographic location in prognosis remains unknown.

## Conclusion

This large series study of pontine infarction revealed the topographic location of unisolated pontine infarction.

## Data Availability

All the data are provided in the manuscript. The potential readers could gain the access to the raw data by email to the YJ via the email address in this manuscript.

## Abbreviations

AF: Atrial fibrillation
BA: Basilar artery
BABD: Basilar artery branch disease
DWI: Diffusion-weighted imaging
LAD: Large artery disease
NIHSS: The National Institutes of Health Stroke Scale
SAD: Small artery disease
TIA: Transient ischemic attack
